# Comparative Analysis of Immune Cell Populations From Two Sampling Techniques of Human Term Decidua Utilizing High‐Parameter Full‐Spectrum Flow Cytometry

**DOI:** 10.1111/aji.70130

**Published:** 2025-07-23

**Authors:** Abigail L. P. Spray, Shahrokh Paktinat, Cara Tobey, Maryam Kalatehjari, Nolawit M. Mulugeta, Michelle Asencio, Sam Rosen, Michael G. Gravett, Lucia Vojtech, Stephen A. McCartney

**Affiliations:** ^1^ Department of Obstetrics and Gynecology University of Washington Seattle Washington USA

**Keywords:** decidua, flow cytometry, immune system, leukocytes, placenta

## Abstract

**Problem:**

The decidua is the interface between the uterus and the fetus. Studying decidual cells can reveal how healthy pregnancies are supported and mechanisms of pregnancy complications. There are two methods of obtaining decidual tissue following delivery. The placental bed can be suctioned following C‐section deliveries, or a thin layer of decidual tissue can be dissected from the placenta. This study aimed to compare immune cell populations obtained using the two methods.

**Method of Study:**

From individuals with scheduled C‐sections, we collected peripheral blood, decidua via vacuum suction of the placental bed, and decidua via dissection of the uterine‐facing side of the placenta. Samples were analyzed using a 22‐color full‐spectrum flow cytometry panel to identify immune cell subsets and functional markers.

**Results:**

The cellular composition of both decidual tissue collection methods were more similar to each other than to peripheral blood. Decidua collected via vacuum suction (Suc. decidua) had more live CD45+ cells. Decidua collected via dissection of the uterine‐facing side of the placenta (Plac. decidua) had significantly higher expression of Helios in CD4+ cells, suggesting more fetal T cells. Both types of decidual samples contained similar levels of Tr1‐like regulatory T lymphocytes expressing LAG3 and CD49b, whereas peripheral blood did not have this cell type.

**Conclusion:**

Collecting decidual tissue using either method resulted in largely similar immune cell populations, suggesting studies are largely comparable regardless of whether samples were collected via suction or placental dissection. This will allow for greater flexibility in sample collection methods.

## 1 Introduction

Immune cells are integral to the decidual–fetal interface, where they regulate cytotrophoblast invasion–a process crucial for proper placental development [1]. Both innate and adaptive immune cells contribute significantly to this interface [2]. Decidual natural killer (dNK) cells are the predominant innate immune cell type, playing roles in vascular remodeling and modulation of trophoblast invasion [3, 4]. Innate lymphoid cells (ILCs) also contribute to the immune environment of the decidua [5]. Myeloid cells, including macrophages and dendritic cells, are other important contributors at the decidual–fetal interface by providing a first line of defense and serving as a crucial link between the innate and adaptive immune systems [6]. Adaptive immune cells, including CD4+ and CD8+ T cells, are involved in antigen recognition and regulation of immune responses to fetal antigens and potential pathogens [7]. Regulatory CD4+ T lymphocytes (Tregs) are essential in maintaining immune tolerance at the interface by modulating the activity of other adaptive immune cells [8]. Dysregulation of any of these immune cell subsets can lead to pregnancy complications such as pre‐eclampsia, preterm birth, chorioamnionitis, recurrent pregnancy loss, and intrauterine growth restriction [9, 10, 11, 12]. Therefore, investigating the various immune cell populations at the decidual–fetal interface could enhance our understanding of the immunological adaptations during normal pregnancy and the pathogenesis of pregnancy‐related disorders.

The decidua is anatomically classified into three regions: (1) decidua basalis: the anchoring site where placental villi invade, with cytotrophoblasts establishing close contact with uterine arteries and immune cells; (2) decidua parietalis: the uterine lining that does not physically participate in placentation and is located away from the placental plate; and (3) decidua capsularis: the layer covering the implanted placental membranes [13]. As the amniotic cavity expands, the decidua capsularis thins and eventually comes into contact with the decidua parietalis, filling the uterine cavity [13].

The decidua basalis is a key site for studying immune‐trophoblast interactions due to its close proximity to the placenta [14]. Various methods have been employed to sample the decidua [15, 16]. One approach involves obtaining biopsies from delivered placentas by dissecting the thin layer of decidua basalis from the uterine‐facing placental plate [15]. Another method entails vacuum suctioning of the placental bed from the uterine cavity following removal of the placenta [16]. Each method has its advantages and limitations. The dissection approach is noninvasive, can be obtained from C‐section and labored deliveries, and provides direct access to the decidua basalis. However, the tissue requires meticulous dissection to remove chorionic villi, which can be time‐consuming, and the samples are often focal rather than representative of the entire placental bed. In contrast, the vacuum suction technique collects tissue from the entire placental bed and is a faster procedure requiring less intensive processing. This method may also yield cells with higher viability due to its rapid nature. However, it is possible that tissue obtained by suction could be contaminated with peripheral blood and amniotic fluid cells, which could be less representative of the decidua basalis because the area of sampling is not limited to the site of placentation and likely includes both decidua basalis and parietalis.

In this study, we investigated differences in immune cell populations obtained from two sampling methods of term decidua from normal pregnancies. To achieve this, we optimized a high‐parameter full‐spectrum flow cytometry panel designed to identify T lymphocytes, B lymphocytes, dNK cells, and ILCs. The panel included several markers for regulatory CD4+ T lymphocyte (Treg) phenotype. Matched peripheral blood mononuclear cells (PBMCs) from each participant were also analyzed to highlight the differences in the immune environment between decidua and peripheral blood.

## 2 Materials and Methods

### 2.1 Human Subjects and Study Approval

The study protocol was approved by the Institutional Review Board at the University of Washington under reference numbers STUDY00001636 and STUDY00011897. Five pregnant individuals were included in this study after providing written informed consent. C‐section procedures were scheduled for all cases. Relevant clinical information for each participant is in Table 1. Peripheral blood samples were collected from each individual immediately prior to the C‐section.

**TABLE 1:** Clinical information for participants.

Participant number	Age	Gestational age	Gravidity	Pregnancy complication(s)	Prior pregnancy complication(s)	General health complication(s)
1	28	39w0d	3	Type I gestational diabetes mellitus (A1GDM)	Pre‐eclampsia with severe features	Class I obesity
2	32	40w1d	3	None	Fetal growth restriction and postpartum hemorrhage	N/A
3	37	39w2d	2	Postpartum hemorrhage in the setting of uterine atony	N/A	Anxiety
4	35	39w1d	3	Vanishing twin syndrome	Intrauterine infection and postpartum hemorrhage	Subclinical hypothyroidism
5	40	38w6d	2	Uterine fibroid and postpartum hemorrhage	N/A	N/A

### 2.2 Decidua Sampling

Decidua was collected via vacuum suctioning performed during the C‐section immediately after delivery of the placenta. The site of placental implantation inside the uterus was identified visually and decidual cells at that site were collected by vacuum aspiration using a sterile disposable mucus trap [16, 17] (Figure S1). Briefly, 5 IU oxytocin was given intravenously to the participant after delivery. The placenta was located by manual palpation, and then separated from the uterine wall. Tissue from the identified placental bed was harvested with the vacuum suction force into a tissue‐sampling container. Although complete exclusion of contaminating cells is not feasible, we are confident that the tissue we processed was primarily decidua basalis.

The collected tissue was immediately transferred to the laboratory. Upon arrival, the sample was washed thoroughly in Dulbecco's phosphate‐buffered saline (DPBS) to remove blood before processing and cell isolation.

Decidua collected via dissection of the placenta occurred in the laboratory. The placenta was transported to the laboratory at room temperature to obtain decidua basalis biopsies. Sampling involved collecting tissue from multiple locations within the cotyledon areas by dissecting the thin decidua basalis layer from the uterine‐facing side of the placenta (Figure S2). The biopsy samples were thoroughly washed in DPBS and further separated to remove any residual material not consistent with decidua basalis prior to enzymatic digestion.

### 2.3 Tissue Digestion and Single Cell Isolation

Both types of decidua samples were minced into small pieces using a scalpel in a 10‐cm plastic Petri dish. The tissue fragments were then enzymatically digested in a cocktail containing 0.5–1 mg/mL of type II collagenase and 20 U/mL of DNase in RPMI medium supplemented with 7.5% fetal bovine serum (FBS). The digestion was carried out at 37°C in a shaking incubator for 30–45 min. The enzymatic reaction was quenched by adding equal volume of RPMI medium containing 10% FBS. To achieve a single‐cell suspension, the digested tissue was filtered consecutively through 70  and 40 µm cell strainers. The strainers were thoroughly washed with complete medium to maximize cell yield. Subsequently, one to two rounds of red blood cell lysis were performed, followed by a final wash and assessment of cell count and viability. The isolated cells were cryopreserved in a freezing medium composed of 10% dimethyl sulfoxide (DMSO) and 90% FBS.

### 2.4 Peripheral Blood Mononuclear Cell Isolation

Whole blood samples were collected in acid citrate dextrose (ACD) tubes and centrifuged at 400×g at room temperature to separate the plasma. Following plasma removal, DPBS was added to the remaining cellular fraction at a 2:1 ratio. Then, the suspension was carefully layered onto a Lymphoprep gradient solution in SepMate tubes (STEMCELL Technologies, 18061, 85450). After centrifugation at 1200×g at room temperature for 10 min, the buffy coat layer, containing PBMCs, was collected and transferred to a clean tube. The cells were washed with DPBS, and concentration and viability were evaluated before cryopreservation.

### 2.5 High Parameter Flow Cytometry

Cells from each sample type, including PBMCs, decidua collected via vacuum suction (Suc. decidua), and decidua collected via dissection of the uterine‐facing side of the placenta (Plac. decidua), were thawed and rested overnight in complete RPMI media. Following incubation, the samples were filtered through a 70‐µm cell strainer, and cell numbers were adjusted to no more than 5 million per sample before staining. The staining was performed in 96‐well plates, starting with a wash in DPBS and staining with a fixable live/dead dye in conjunction with Fc‐receptor blocking (BioLegend Human TruStain FcX, 422302). Extracellular staining was conducted in FACS buffer (2% FBS in DPBS) containing Brilliant Stain Buffer Plus (BD Biosciences, 566385). After extracellular staining, the cells were fixed and permeabilized using the FOXP3 Fixation/Permeabilization Kit (Invitrogen, 00‐5523‐00), followed by intracellular and intranuclear staining. The procedure concluded with fixation in 4% paraformaldehyde (PFA) solution (Fisher Scientific, 50‐980‐491) in DPBS and resuspension of the cells in FACS buffer for flow cytometry analysis. Single‐stained cell or bead (Invitrogen, 01‐2222‐42) controls were prepared simultaneously using the same procedure as the samples. Flow cytometry was conducted on a full‐spectrum, five‐laser Cytek Aurora instrument at the University of Washington Cell Analysis Facility. Table S1 includes a detailed description of the antibodies used in the panel.

### 2.6 Flow Cytometry Data Cleanup for Dimensionality Reduction Analysis

Data were unmixed in SpectroFlo software (Cytek Technologies, version 3.2.1) and then exported and uploaded to FlowJo software (BD Biosciences, version 10.10.0). Data cleanup was performed following a published protocol with minor modifications [18]. Gating was done in FlowJo to exclude dead cells, debris, doublets, and nonleukocyte cells (CD45−). Downsampling was carried out based on the sample with the smallest number of cells across the entire sample set (30,191 cells) and the.fcs files were exported back into the SpectroFlo software to merge each sample type into a single.fcs file. Merged files were uploaded into FlowJo and concatenated into a new.fcs file containing all sample types. Gating was used to exclude supernegative and superpositive events. Scale adjustment was performed before proceeding to unsupervised dimensionality reduction analysis. The Uniform Manifold Approximation and Projection (UMAP) algorithm was applied to the concatenated file, followed by clustering using the FlowSOM algorithm, with clusters explored using the FlowJo Cluster Explorer plugin.

### 2.7 Statistical Analysis

Statistical analyses were performed using GraphPad Prism 9, applying two‐tailed paired *t*‐tests for comparisons between sample types. Significance levels were denoted as follows: *p ≤ 0.05, **p ≤ 0.01, and ***p ≤ 0.001. Any *p* value below 0.05 was deemed to indicate statistical significance.

## 3 Results

### 3.1 Major Immune Populations Present in Decidua Tissue

We characterized the immune cells isolated from three sample types, including PBMCs, decidua basalis dissected from placenta (Plac. decidua), and decidua collected via suction (Suc. decidua) from five participants. Table 1 shows the relevant clinical information for participants.

**FIGURE 1:**
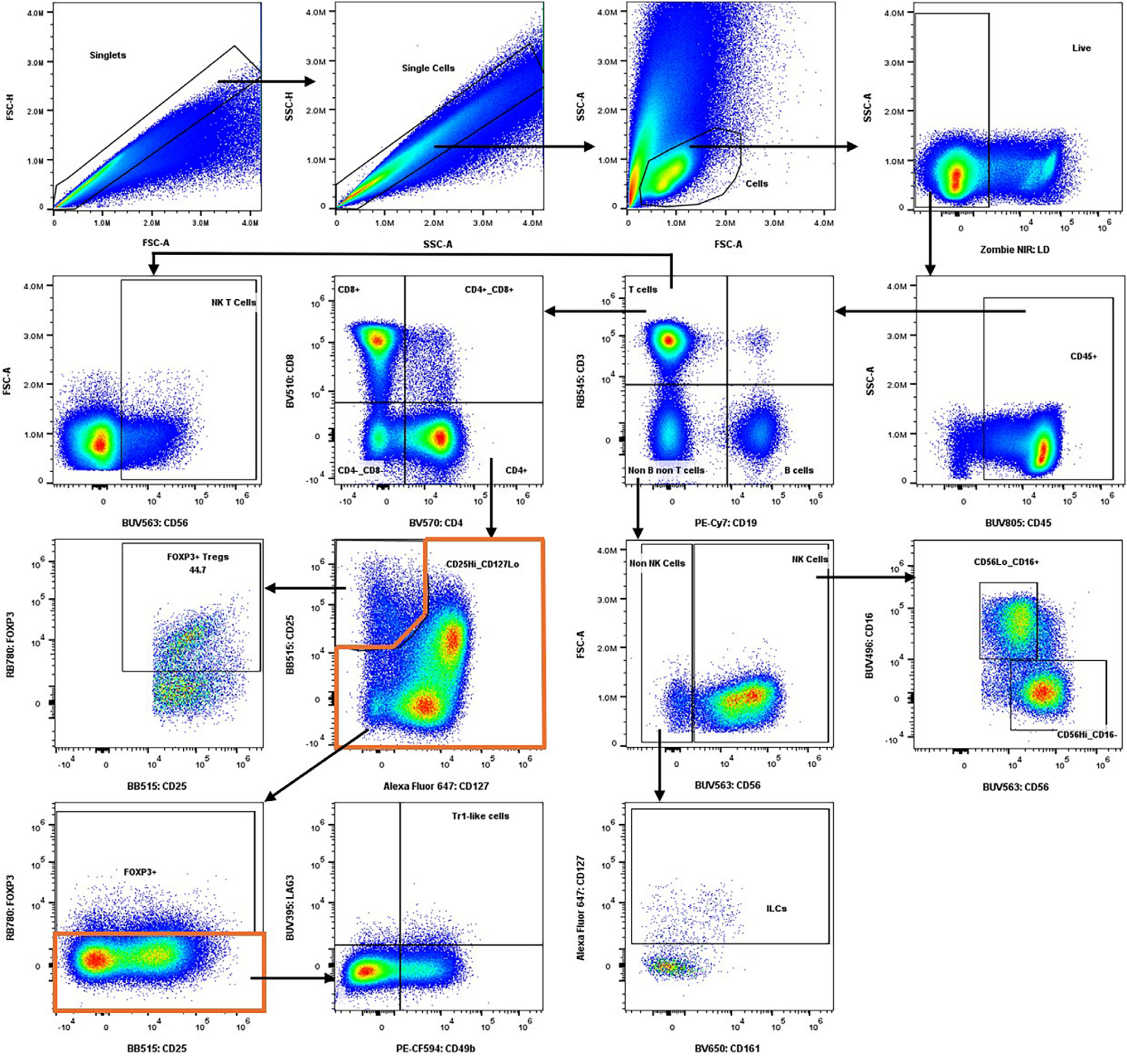
Gating strategy to identify the major immune cell types. Gating shows live leukocytes including T lymphocytes, B lymphocytes, natural killer (NK) cells, innate lymphoid cells (ILCs), regulatory T lymphocytes (Tregs), and type 1 regulatory‐like T lymphocytes.

Major immune cell types were identified utilizing a 22‐color full‐spectrum flow cytometry panel (Figure 1). Table 2 summarizes the antigens included in the panel with their target populations and functions. Live leukocytes (CD45+) cells were gated after eliminating doublets. We then gated on B lymphocytes (CD3−CD19+), T lymphocytes (CD3+CD19−), and non‐B or non‐T lymphocytes (CD3−CD19−). Natural killer (NK) cells were defined as CD56+ from the non‐B non‐T lymphocyte population. Within the NK cells, we identified CD56HiCD16− and CD56LoCD16+ populations. ILCs were classified as either CD127+CD161+ or CD127+CD161− within the CD56− gate. CD4+ T lymphocytes and CD8+ T lymphocytes were identified in the T lymphocyte gate. Regulatory T lymphocytes (Tregs) were defined within the CD4+ lymphocytes as CD25HiCD127LoFOXP3+. An additional population of regulatory CD4+ T lymphocytes falling outside of the CD25HiCD127Lo gate were identified as type 1 regulatory‐like (Tr1) T lymphocytes (FOXP3‐CD49b+LAG3+).

**TABLE 2:** Antigens included in flow cytometry panel with target population.

Antigen	Target population and/or function
**LAG3**	Inhibitory marker expressed on regulatory T lymphocytes that can downregulate T‐cell receptor signal transduction and T‐cell clonal expansion
**CD16**	Marker expressed on natural killer cells that functions in antibody‐dependent cellular cytotoxicity and other antibody‐dependent responses
**CD56**	Lineage marker for natural killer cells
**Ki‐67**	Marker expressed in proliferating cells
**CD45**	Lineage marker for leukocytes including lymphocytes, natural killer cells, innate lymphoid cells, etc.
**PD‐1**	Expressed on T lymphocytes and its interaction with PD‐Ligands inhibits T lymphocyte proliferation and cytokine secretion
**CD8**	Lineage marker for T lymphocytes and functions as a co‐receptor with MHC class I‐restricted T‐cell receptors
**CD4**	Lineage marker for T lymphocytes and functions as a co‐receptor with MHC class II‐restricted T‐cell receptors
**CD161**	Lineage marker used for identification of innate lymphoid cells (ILC2)
**CTLA‐4**	Expressed in T lymphocytes, including regulatory T lymphocytes, and functions as a component of negative regulatory signaling to T cells
**CD94**	Expressed on natural killer cells, interacts with NKG2 receptors to form receptors for HLA class I molecules, and plays a role in regulating cellular adhesion and activation
**CD25**	Marker of activation, highly expressed on T regulatory lymphocytes, and acts as a receptor for Interleukin‐2
**CD3**	Lineage marker for T lymphocytes and functions as a component of the T‐cell receptor complex
**TIGIT**	Expressed on T lymphocytes, including regulatory T lymphocytes, that can downregulate T‐cell receptor‐mediated T‐cell activation and proliferation
**CD49a**	Expressed on natural killer cells (and T lymphocytes) and functions as a cellular adhesion molecule and can co‐stimulate T‐cell proliferation and cytokine production
**FOXP3**	Expressed in regulatory T lymphocytes, and functions as a transcriptional regulator of the NFAT and NF‐κB pathways
**Helios**	Expressed in regulatory T lymphocytes, and functions as a transcriptional regulator contributing to phenotypic stability of regulatory T cells
**CD49b**	Expressed on T lymphocytes and functions as a cell‐cell adhesion receptor
**CD19**	Lineage marker for B lymphocytes and functions as a component of the B‐cell receptor complex
**CD127**	Expressed on T lymphocytes and other lymphoid cells, such as innate lymphoid cells, and functions as a receptor for Interleukin‐7
**IL10**	Expressed in T and B lymphocytes as a multifunctional cytokine that can downregulate immune and proinflammatory responses

**FIGURE 2:**
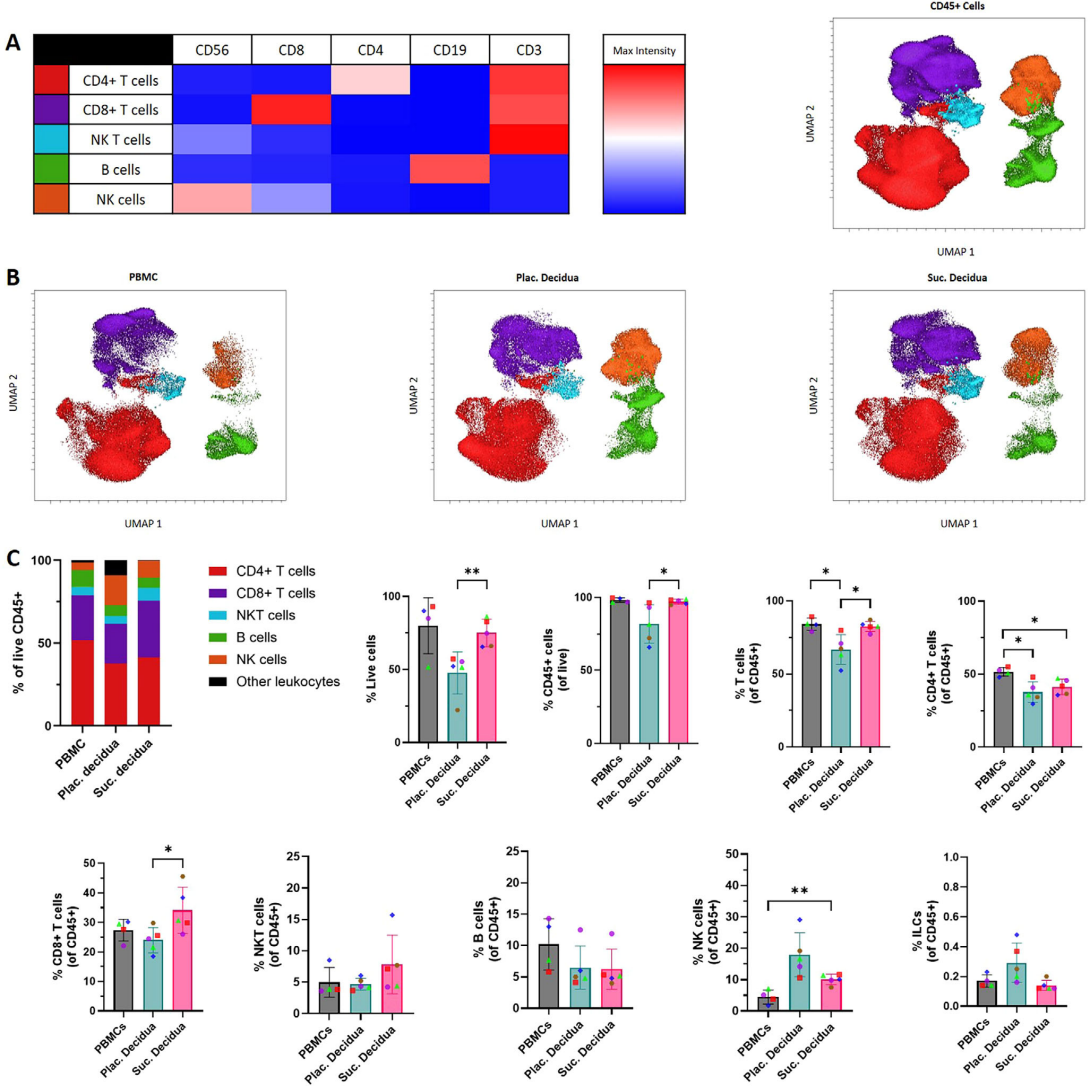
Overview of immune cell types. (A) Uniform Manifold Approximation and Projection (UMAP) and FlowSOM clustering containing data from all participants and all sample types. (B) UMAP for each sample type. (C) Bar graphs showing immune cell type frequencies in the live leukocyte (CD45+) population. Paired samples are represented by the same color/shape. A two‐tailed paired *t*‐test was used to compare frequencies between sample types. Significance levels are indicated as follows: *p ≤ 0.05 and **p ≤ 0.01.

Dimensionality reduction analysis using the UMAP algorithm and the FlowSOM algorithm identified major clusters of immune cells in the combined peripheral blood and decidua samples. Figure 2A depicts the clusters in one UMAP containing data from all participants and all sample types. Cluster 1 was defined as CD4+ T lymphocytes due to the high expression of CD3 and CD4. Cluster 2 was defined as CD8+ T lymphocytes due to the high expression of CD3 and CD8. Cluster 3 was defined as NK T lymphocytes due to the high expression of CD3 and the expression of CD56. Cluster 4 was defined as B lymphocytes due to the high expression of CD19. Cluster 5 was defined as NK cells due to the expression of CD56. Figure 2B highlights the differences in the density of the clusters in each sample type. Notably, the NK and NKT populations were reduced in PBMC compared to decidua. Following this analysis, we performed 2D gating to further characterize the immune subsets and compare the frequencies of these subsets between the two decidua sampling methods (Figure 2C). The Suc. decidua had higher frequencies of live cells (*p* ≤ 0.01), CD45+ cells (*p* ≤ 0.05), and T lymphocytes (*p* ≤ 0.05) compared to the Plac. decidua. Additionally, the Plac. decidua had lower frequencies of T lymphocytes than the PBMCs (*p* ≤ 0.05). Lower frequencies of CD4+ T lymphocytes were detected in both decidua types compared to PBMCs (*p* ≤ 0.05). The Suc. decidua had a higher frequency of CD8+ T lymphocytes than the Plac. decidua. Plac. decidua had trending higher levels of NK cells, but this did not reach statistical significance. Both decidua types had comparable frequencies of B lymphocytes, CD4+ T lymphocytes, and ILCs. The Suc. decidua samples had a significantly higher frequency of NK cells compared to the PBMCs (*p* ≤ 0.01).

### 3.2 T Lymphocyte Profiling

**FIGURE 3:**
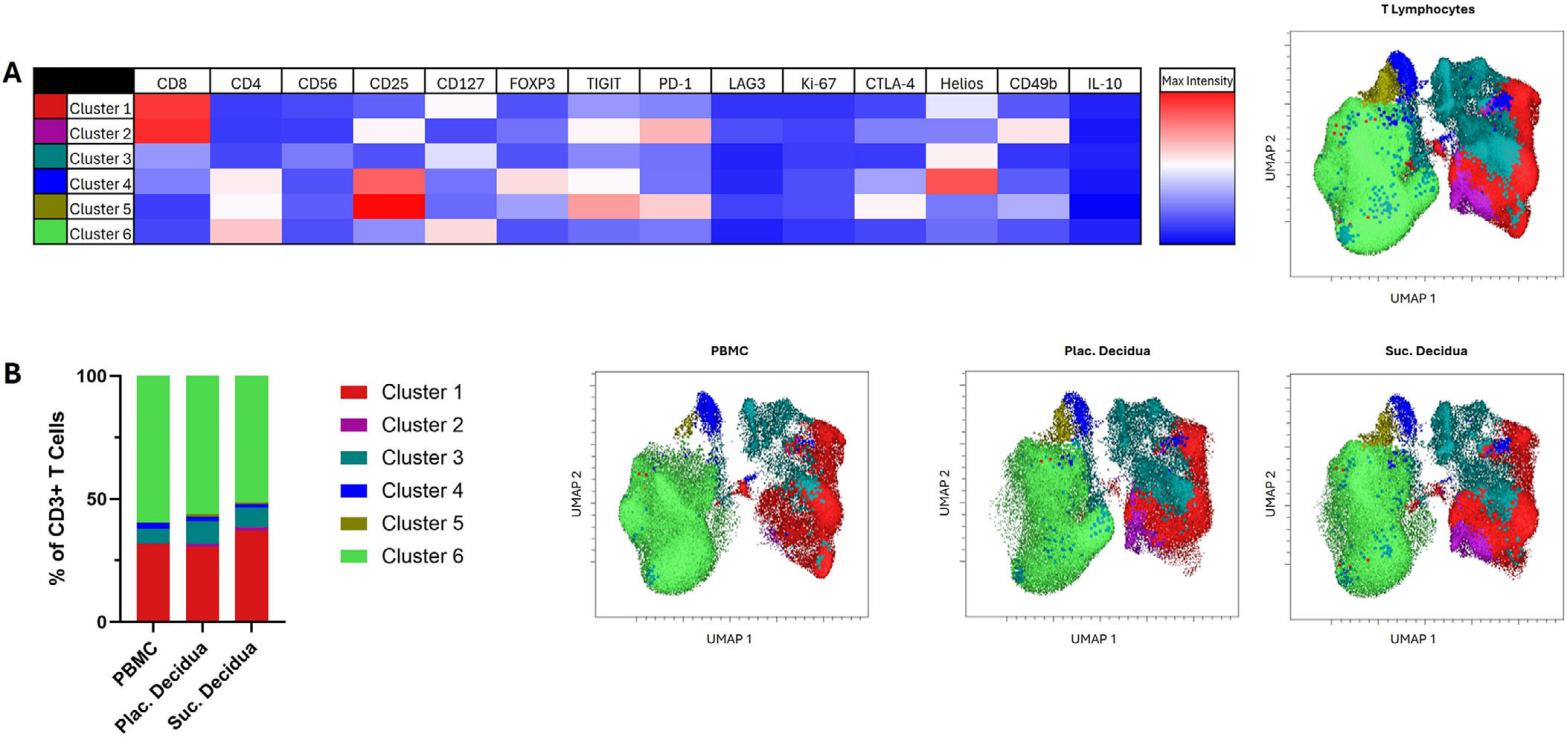
Overview of T lymphocyte populations. (A) Uniform Manifold Approximation and Projection (UMAP) and FlowSOM clustering containing data from all participants and all sample types. Scaling for the heat map is across all markers. (B) Stacked bar graph and UMAPs illustrating the frequencies of clusters in each sample type.

We next wanted to look in more detail into the largest population, which was CD3+ T lymphocytes. To do so, we analyzed the CD3+ population across sample types using UMAP and FlowSOM. This showed six clusters based on their variable expression of lymphocyte‐associated markers (Figure 3A,B). The largest clusters, regardless of sample type, were cluster 1 and cluster 6. Cluster 1 was identified as CD8+ T lymphocytes expressing CD127 and Helios. Cluster 6 had a higher frequency compared to cluster 1 and was identified as CD4+ T lymphocytes expressing CD127. The frequencies of these clusters were comparable across the sample types. Cluster 3 was identified as a population of NK T lymphocytes (CD3+CD56+). The frequency of cluster 3 was similar across sample types, but was slightly higher in the decidua samples. Cluster 4 was identified as regulatory CD4+ T lymphocytes (CD25hiCD127loFOXP3+). This cluster also had the highest expression of Helios. Cluster 4 was found at a similar frequency in the decidua sample types. Cluster 2 was a small population of CD8+ T lymphocytes expressing CD25 and TIGIT. The cells in this cluster were also highly expressing PD‐1 and CD49b. Cluster 2 was found at a higher frequency in both decidua sample types (Plac. decidua, 1.1%; Suc. decidua, 1.4%) compared to PBMC (0.1%). Cluster 5 was a small population of CD4+ T lymphocytes highly expressing CD25, TIGIT, and PD‐1. This cluster was found at a higher frequency in both decidua sample types (Plac. decidua, 1.0%; Suc. decidua, 0.8%) compared to PBMC (0.1%).

**FIGURE 4:**
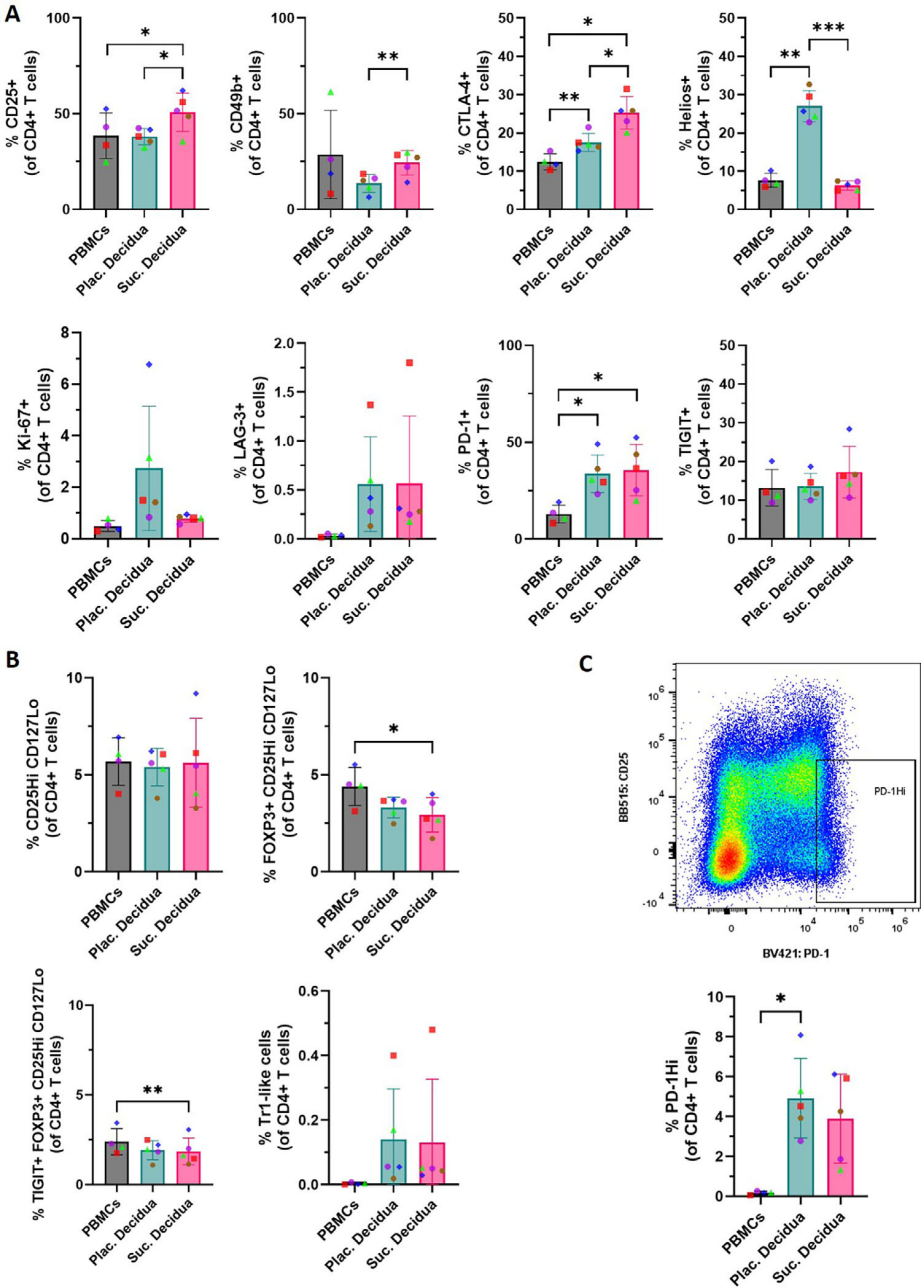
Expression pattern of lymphocyte‐associated markers in CD4+ T lymphocytes. (A) Comparison of lymphocyte markers in CD4+ T lymphocytes and (B and C) regulatory CD4+ T lymphocytes. Paired samples are represented by the same color/shape. A two‐tailed paired *t*‐test was used to compare frequencies between sample types. Significance levels are indicated as follows: *p ≤ 0.05, **p ≤ 0.01, and ***p ≤ 0.001.

We analyzed the CD4+ T lymphocytes by 2D gating following the UMAP and FlowSOM analysis (Figure 4A). There was slightly greater expression of CD25 (*p* ≤ 0.05), CD49b (*p* ≤ 0.01), and CTLA‐4 (*p* ≤ 0.05) in the Suc. decidua samples compared to the Plac. decidua. The Plac. decidua samples had higher levels of Helios expression (*p* ≤ 0.001) and trending higher expression of Ki‐67 compared to the Suc. decidua samples. Both tissues had similar expression of LAG‐3, PD‐1, and TIGIT markers. CTLA‐4 and PD‐1 expression was greater in both sample types compared to the PBMC samples. Figure 4B shows the comparison of regulatory CD4+ T lymphocyte (Tregs) phenotypes between the sample types. There were only small differences in Treg populations between decidua sample types as well as compared to PBMC. Specifically, there were no significant differences in the frequency of CD25HiCD127Lo CD4+ T lymphocytes across sample types. The frequencies of FOXP3+ Tregs and TIGIT+FOXP3+ Tregs were not significantly different between the decidua types, but Suc. decidua had lower frequencies than the PBMCs (*p* ≤ 0.05 and **p ≤ 0.01, respectively). The most notable difference in regulatory T cells was that Suc. decidua and Plac. decidua had detectable Tr1‐like populations, whereas PBMCs lacked this population. The decidua samples also showed a unique population of PD‐1Hi CD4+ T lymphocytes previously described in the literature [19] which was absent in the PBMCs (Figure 4C).

### 3.3 NK Cell Phenotypes in the Decidua

**FIGURE 5:**
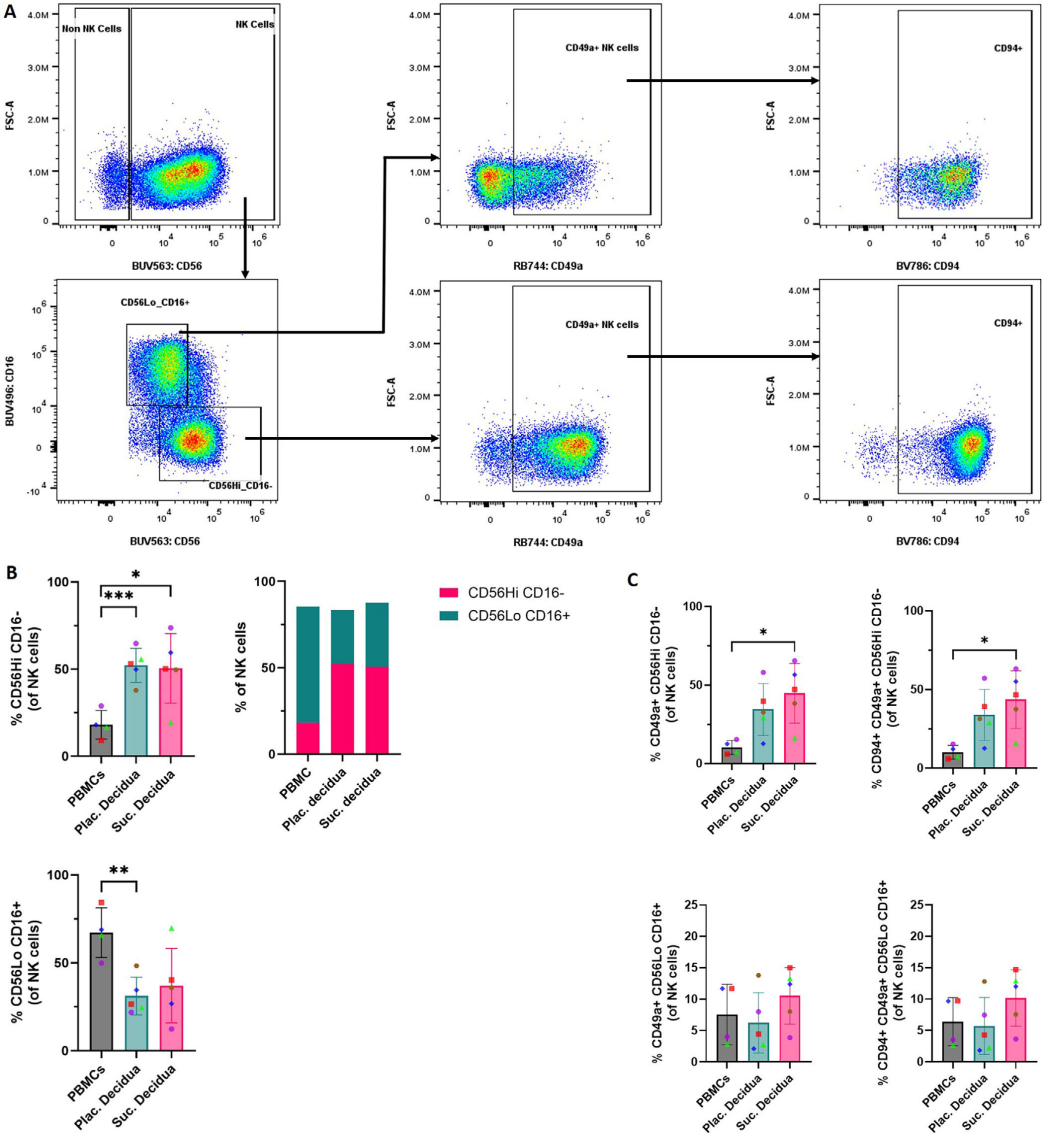
Subsets of natural killer (NK) cells (CD56+) across sample types. (A) Gating strategy to define tissue‐resident (CD49a+) populations in natural killer cell subpopulations based on expression level of CD56 and CD16. (B) Frequencies of two natural killer cell subpopulations in individual sample types. Stacked bar graph represents composition of the natural killer cell subpopulations in each sample type. (C) Bar graph comparison of tissue‐resident natural killer cells, defined as CD49a+, and expression level of CD94. Paired samples are represented by the same color/shape. A two‐tailed paired *t*‐test was used to compare frequencies between sample types. Significance levels are indicated as follows: *p ≤ 0.05, **p ≤ 0.01, and ***p ≤ 0.001.

Finally, we looked more closely at NK cells, due to the importance of NKs found in the decidua during pregnancy. Figure 5A shows the gating strategy for identification of major NK cell subsets, including CD56LoCD16+ NK cells and CD56HiCD16− NK cells. Further gating was performed to identify CD49a+ NK cells expressing CD94 in both subsets. As expected, both Suc. decidua and Plac. decidua had higher frequencies of CD56HiCD16− NK cells compared to the PBMCs (Figure 5B, *p ≤ 0.05 and ***p ≤ 0.001, respectively). CD56LoCD16+ NK cell frequencies were lower in both decidua types compared to PBMCs. The composition of these two subsets of NK cells in each sample type is represented in the stacked bar graph of total NK cells. Further, expression of tissue residency markers (CD49a and CD94) was much higher in the decidua samples than in PBMC, but there was little difference between the two decidua sampling methods (Figure 5C). Thus, as expected, NK cells from the decidua have a much more tissue‐resident phenotype than those from the blood. There were no major differences between NK cells from the two decidua sampling types.

## 4 Discussion

Analyzing the immune cell composition of the decidua provides valuable insights into the regulation of trophoblast invasion and decidual–fetal immune interactions. There are multiple methods for collection of human decidua and placenta tissues. To the best of our knowledge, the comparison of immune cell populations obtained by these individual methods has not been previously reported. This study aimed to compare two methods of collecting decidua tissues from human term pregnancies for isolation of immune cells: vacuum suction of the placental bed (Suc. decidua) and dissection of the uterine‐facing placental plate (Plac. decidua).

In 1999, Staff et al. introduced the isolation of decidua by vacuum suction of the placental bed [17]. The suction method has been used to investigate endoplasmic reticulum stress in complicated pregnancies [20], transcriptional profiling of the decidua in pre‐eclampsia [21], and cholesterol crystal accumulation and NLRP3 inflammasome expression in the decidua in pre‐eclamptic pregnancies [22]. There are a few publications using the suction method in studies of decidual immune cell types including NK cells [23], regulatory T lymphocyte and dendritic cell [24], and myeloid‐derived suppressor cells [25]. The dissection method from the basal plate of the placenta has been described in several publications [26, 27]. Ikumi et al. comprehensively described the placental dissection method for isolation of decidual leukocytes [15]. The dissection method from the basal plate of the placenta has frequently been utilized in immunophenotyping studies of decidual cell types including macrophages [26, 28, 29, 30], CD8+ T lymphocytes [31, 32, 33], NK cells [34], and regulatory CD4+ T lymphocytes [19]. The phenotypes of immune cells have not been directly compared between Suc. decidua and Plac. decidua, and both methods have strengths and weaknesses. The dissection method can be utilized with any placenta, but it requires more technical training, and can be more time consuming. The tissue obtained using the dissection method might also result in contamination with fetal derived cells. The suction method is faster, but requires a C‐section delivery. The biopsies obtained utilizing the dissection method are almost entirely decidua basalis. A limitation of our study is that tissue collected utilizing the suction method could include both decidua basalis and parietalis, which have been shown to have different phenotypic and activation profiles, with higher activated and co‐inhibitory phenotypes in the decidua parietalis [35]. We cannot rule out that the small differences between methods we observed are due to the presence of decidua parietalis cells in the Suc. samples, or some fetal cells co‐purified in the Plac. samples. By using samples from the same individual donors, we were able to directly compare the decidua collection methods. A primary aim of this study was to demonstrate whether the immune cell populations isolated from decidua are broadly similar regardless of which method is used–and indeed we found that to be the case.

We began our analysis with broadly defining the major cell populations known to play a biologically important role at the decidual–fetal interface. These included B lymphocytes, NK cells, ILC, and regulatory and nonregulatory T lymphocytes. We used dimensionality reduction to identify and compare the major cell populations within each sample type (Figure 2A,B), which showed that the two methods resulted in broadly similar leukocyte populations. This analysis identified five distinct populations, including CD4+ T lymphocytes, CD8+ T lymphocytes, NKT lymphocytes, B lymphocytes, and NK cells. These populations were further investigated using 2D gating (Figure 2C). We observed significantly higher recovery of live cells, CD45+ cells, and CD45+ T cells in the Suc. method compared to the Plac. method. The additional processing steps and time between delivery and cell isolation in the dissection method could be an explanation for the lower frequency of viable cells in Plac. decidua. Another possible explanation for the lower viability in Plac. decidua is the natural degradation of cells from the placenta at parturition. Other notable differences included lower frequencies of CD45+ leukocytes, CD3+ T lymphocytes, and CD8+ T lymphocytes in the Plac. decidua compared to the Suc. decidua (Figure 2C), potentially due to nonleukocyte placental cells remaining in the Plac. decidua samples. Additionally, both decidua isolation methods contained comparable frequencies of B lymphocytes, CD4+ T lymphocytes, and ILCs. The frequency of NK cells appears to be trending higher in Plac. decidua compared to Suc. decidua. This may be attributed to the proximity of the Plac. decidua to the trophoblast cells and the interactions between trophoblast cells and NK cells [36].

We next analyzed the largest leukocyte population (CD3+ T lymphocytes) in greater detail. To visualize overall population dynamics between the sample types, we first did dimensionality reduction analysis of the CD3+ T lymphocytes, which revealed six distinct populations (Figure 3A,B). There were minimal differences in the frequency of these clusters between the Suc. decidua and Plac. decidua. This demonstrates that the tissues collected with either sampling method contained comparable populations of CD3+ T lymphocytes. Additionally, the decidua samples contained two clusters that were detected at much higher frequencies compared to the PBMC. This included cluster 2 which was composed of CD8+ T lymphocytes expressing CD25 and TIGIT, and cluster 5 which was composed of CD4+ T lymphocytes highly expressing CD25, TIGIT, and PD‐1.

As the presence of regulatory T cells in the decidua is essential for maintaining pregnancy, we focused more on regulatory functional markers within CD4+ T lymphocytes. 2D gating analysis revealed a greater expression of CD25, CD49b, and CTLA‐4 in the Suc. decidua compared to the Plac. decidua (Figure 4A). A previous study reported higher frequencies of CD4+CD25+ T lymphocytes found in the decidua parietalis compared to the decidua basalis [37]. Therefore, a higher frequency of CD4+CD25+ T lymphocytes in the Suc. decidua could be a result of the potential mixed composition of decidua basalis and parietalis. Another difference between the tissues was that the Plac. decidua had higher expression of Helios and trending higher expression of Ki‐67 in the CD4+ compartment (Figure 4). Helios is a transcription factor that is expressed in a large fraction of Tregs, and also by fetal naïve CD4+ T cells, where it plays a role in differentiating fetal CD4+ T cells preferentially into regulatory T cells [38]. Ki‐67 is a marker of proliferative potential and is highly expressed by trophoblast cells. These findings suggest that the Plac. decidua method results in some level of contamination with fetal immune cells. It is technically challenging to distinguish fetal and decidual immune cells and we did not attempt to quantify the levels of fetal cell in each sample in this study. For studies where pure decidual cell samples are required, it may be advisable to use the Suc. method.

Further analysis of regulatory CD4+ T lymphocyte subsets showed no differences between the decidua sample types. Specifically, both decidua sample types had similar levels of FOXP3+ Tregs, TIGIT+ Tregs, Tr1‐like lymphocytes, and PD‐1Hi lymphocytes. Both samples had PD‐1 high cells and Tr1‐like cells (CD49b+LAG3+), previously described in decidual samples [19], whereas PBMCs lacked this cell type (Figure 4B,C). Similar results were observed in the NK cell subsets, with both decidual sample types having comparable NK cell populations (Figure 5). In particular, the decidua samples showed high levels of tissue‐resident (CD49a+ cells) and CD56HiCD16− NK cells, whereas peripheral blood had low levels of these cells (Figure 5B). Within NK cells, CD94, a uterine NK cell marker [39], was expressed at similarly high levels in both types of decidual samples, but detected at low levels in peripheral blood NK cells (Figure 5C).

Altogether, our findings indicate that both decidua sampling methods are much more similar to each other than to PBMCs, though Plac. samples may have more fetal cells. This comparison validates that both methods of decidua collection will result in largely similar immune cell subsets, increasing translatability across studies and broadening possible study designs to overcome logistical and technical challenges in collecting and studying decidual tissue.

## Ethics Statement

The authors confirm that the ethical policies of the journal, as noted on the journal's author guidelines page, have been adhered to and the appropriate ethical review committee approval has been received. The study conformed to the US Federal Policy for the Protection of Human Subjects.

## Conflicts of Interest

The authors declare no conflicts of interest.

## Supporting information




**Supporting Figure 1:** aji70130‐sup‐0001‐Figures_1.docx

## Data Availability

The data that support the findings of this study are available from the corresponding author upon reasonable request.
