# Supplementary material for: Comparative Analysis of Immune Cell Populations From Two Sampling Techniques of Human Term Decidua Utilizing High‐Parameter Full‐Spectrum Flow Cytometry
Source: Am J Reprod Immunol. 2025 Jul 23;94(2):e70130. doi: 10.1111/aji.70130 (PMC12284915; doi:10.1111/aji.70130)
Supplement: Supplementary file 1 — Supporting Figure 1: aji70130‐sup‐0001‐Figures_1.docx [file AJI-94-e70130-s001.docx]

**Supplemental Information**


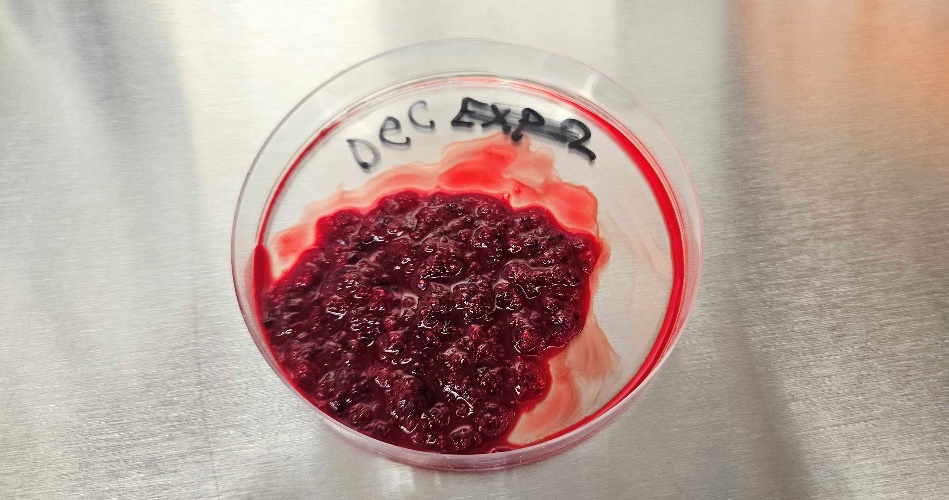


**Supplemental Figure 1.** Image of decidua collected via vacuum suctioning.


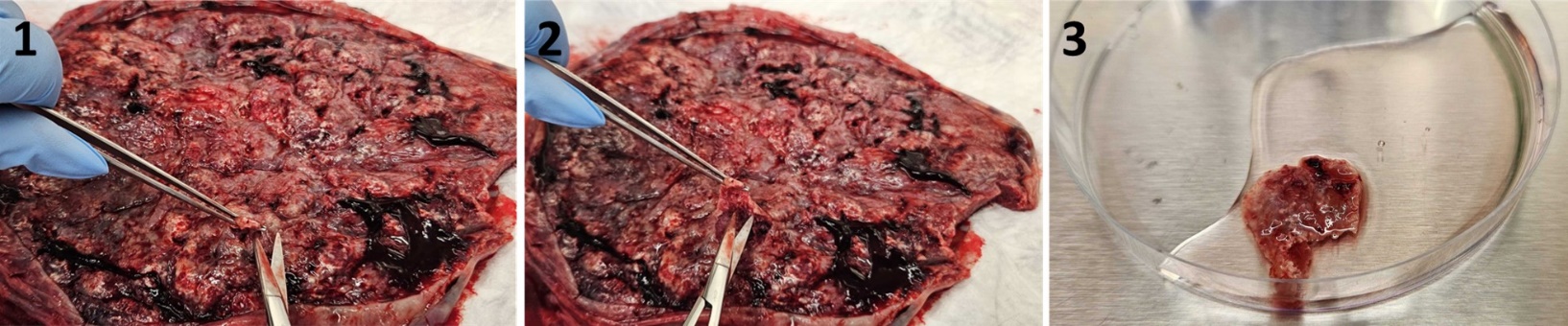


**Supplemental Figure 2.** Images demonstrating the process of obtaining decidua basalis biopsies from the placenta.

**Supplemental Table 1.** 22-color flow cytometry panel used for characterization of immune cell populations in peripheral blood mononuclear cells (PBMC) and decidua tissue samples

| **22c Phenotyping Panel - OVERVIEW** | | | | | |  |  |  |  |  |
| --- | --- | --- | --- | --- | --- | --- | --- | --- | --- | --- |
|  | **Laser** | **Fluorophore** | **Antigen** | **Clone** | **Vendor** | **Catalog #** | **Host** | **Single Stained Control** | **Cocktail** | **Titer** |
| 1 | 355nm | BUV395 | LAG3 | T47-530 | BD Biosciences | 569247 | Mouse | Beads | Extracellular | 1 to 40 |
| 2 |  | BUV496 | CD16 | 3G8 | BD Biosciences | 612945 | Mouse | Beads | Extracellular | 1 to 200 |
| 3 |  | BUV563 | CD56 | NCAM16.2 | BD Biosciences | 612928 | Mouse | Beads | Extracellular | 1 to 160 |
| 4 |  | BUV737 | Ki-67 | B56 | BD Biosciences | 567130 | Mouse | Beads | Intracellular/Intranuclear | 1 to 320 |
| 5 |  | BUV805 | CD45 | HI30 | BD Biosciences | 612891 | Mouse | Beads | Extracellular | 1 to 500 |
| 6 | 405nm | BV421 | PD-1 | EH12.1 | BD Biosciences | 565935 | Mouse | Beads | Extracellular | 1 to 160 |
| 7 |  | BV510 | CD8 | RPA-T8 | BD Biosciences | 563256 | Mouse | Beads | Extracellular | 1 to 80 |
| 8 |  | BV570 | CD4 | RPA-T4 | BioLegend | 300533 | Mouse | Beads | Extracellular | 1 to 80 |
| 9 |  | BV650 | CD161 | DX12 | BD Biosciences | 563864 | Mouse | Beads | Extracellular | 1 to 320 |
| 10 |  | BV711 | CTLA-4 | BNI3 | BioLegend | 369631 | Mouse | Beads | Intracellular/Intranuclear | 1 to 640 |
| 11 |  | BV786 | CD94 | HP-3D9 | BD Biosciences | 743953 | Mouse | Beads | Extracellular | 1 to 40 |
| 12 | 488nm | BB515 | CD25 | 2A3 | BD Biosciences | 564467 | Mouse | Beads | Extracellular | 1 to 320 |
| 13 |  | RB545 | CD3 | SK7 | BD Biosciences | 756481 | Mouse | Cells | Extracellular | 1 to 320 |
| 14 |  | BB700 | TIGIT | 741182 | BD Biosciences | 747846 | Mouse | Beads | Extracellular | 1 to 320 |
| 15 |  | RB744 | CD49a | TS2/7 | BD Biosciences | 757021 | Mouse | Beads | Extracellular | 1 to 640 |
| 16 |  | RB780 | FOXP3 | 259D/C7 | BD Biosciences | 568682 | Mouse | Beads | Intracellular/Intranuclear | 1 to 40 |
| 17 | 532nm | PE | Helios | 22F6 | BD Biosciences | 563801 | Hamster | Cells | Intracellular/Intranuclear | 1 to 640 |
| 18 |  | PE-CF594 | CD49b | 12F1 | BD Biosciences | 564121 | Mouse | Beads | Extracellular | 1 to 320 |
| 19 |  | PE-Cy7 | CD19 | SJ25C1 | BD Biosciences | 557835 | Mouse | Beads | Extracellular | 1 to 320 |
| 20 | 628nm | AF647 | CD127 | HIL-7R-M21 | BD Biosciences | 560905 | Mouse | Beads | Extracellular | 1 to 20 |
| 21 |  | R718 | IL10 | JES3-19F1 | BD Biosciences | 567246 | Rat | Beads | Intracellular/Intranuclear | 1 to 160 |
| 22 |  | Zombie NIR | Live/Dead | NA | BioLegend | 423105 | NA | Cells | Live/Dead | 1 to 1600 |
